# The UBC9/SUMO pathway affects E-cadherin cleavage in HPV-positive head and neck cancer

**DOI:** 10.3389/fmolb.2022.940449

**Published:** 2022-08-12

**Authors:** Maria Elisa Sabatini, Micaela Compagnoni, Fausto Maffini, Claudia Miccolo, Fabio Pagni, Mariano Lombardi, Virginia Brambilla, Daniela Lepanto, Marta Tagliabue, Mohssen Ansarin, Simona Citro, Susanna Chiocca

**Affiliations:** ^1^ Department of Experimental Oncology, IEO, European Institute of Oncology IRCCS, IEO Campus, Milan, Italy; ^2^ Division of Pathology, IEO, European Institute of Oncology, IRCCS, Milan, Italy; ^3^ Department of Medicine and Surgery, Pathology, San Gerardo Hospital, University of Milano-Bicocca, Monza, Italy; ^4^ Division of Otolaryngology Head and Neck Surgery, IEO, European Institute of Oncology IRCCS, Milan, Italy

**Keywords:** head and neck cancer, head and neck squamous cell carcinoma, HPV, SUMO, Ubc9, E-cadherin

## Abstract

Functional loss of E-cadherin is frequent during tumor progression and occurs through a variety of mechanisms, including proteolytic cleavage. E-cadherin downregulation leads to the conversion of a more malignant phenotype promoting Epithelial to Mesenchymal Transition (EMT). The UBC9/SUMO pathway has been also shown to be involved in the regulation of EMT in different cancers. Here we found an increased expression of UBC9 in the progression of Head and Neck Cancer (HNC) and uncovered a role for UBC9/SUMO in hampering the HPV-mediated E-cadherin cleavage in HNC.

## Introduction

Head and Neck Cancer (HNC) is the sixth most common cancer worldwide (Bray et al., 2018) with a 5-year survival of 50%–60%. It arises in the mucosal epithelium of the oral cavity, oropharynx, larynx, or hypopharynx. Oral cavity and larynx cancers are generally associated with the use of tobacco and excessive alcohol consumption, whereas oropharynx cancers are increasingly attributed to infection with human papillomavirus (HPV), primarily HPV16. Thus, HNC can be classified into HPV-negative and HPV-positive HNC and, whereas the incidence of HPV-negative tumors is slowly declining, those of HPV-positive is rapidly increasing in the western countries. Despite HPV-positive oropharyngeal squamous cell carcinoma (OPC) has been shown to have a more favorable prognosis compared to HPV-negative HNC, HPV-OPC often tends to exhibit large cystic metastases in the cervical lymph nodes, with a clinically and radiologically occult primary tumor(Paver et al., 2020). High-risk HPVs affect numerous cellular pathways, trying to complete its life-cycle and to induce persistent infection. One of the affected pathways is the Epithelial to Mesenchymal transition (EMT). EMT is a physiological process during embryonic development, wound closure, and fibrosis, although in solid tumors, EMT increases their invasiveness and metastatic activity (Yang et al., 2020). Many pathogens affect this pathway targeting in particular E-cadherin. E-cadherin is a cell-cell adhesion transmembrane molecule with a crucial role in cell adhesion and in maintaining an epithelial cell phenotype; thus, loss of E-cadherin promotes EMT. It has been shown that HPV reduces E-cadherin expression by epigenetic and transcriptional regulation (Laurson et al., 2010), whereas other pathogens, such as *Helicobacter pylori*, promote loss of E-cadherin by inducing its shedding (Weydig et al., 2007). Another pathway affected by HPV is the SUMO pathway, and we recently published that the unique SUMO-conjugating enzyme, UBC9 increases its expression in cervical cancer and in HNC progression (Mattoscio, 2015; Mattoscio et al., 2017). UBC9 has been shown to mainly target nuclear proteins, but also membrane proteins require binding to UBC9 to maintain their localization (Collec et al., 2007; Xiong et al., 2017). Here we show that UBC9 interacts with E-cadherin and the loss of SUMO increases E-cadherin cleavage in HPV-positive HNC cell lines. HPV infection causes increased E-cadherin cleavage and degradation, which affects E-cadherin localization at the plasma membrane.

## Materials and methods

### Cell culture and tissue samples

HPV-positive and negative HNC cell lines were obtained from different sources (Brenner et al., 2010), as already described in our previous studies (Citro et al., 2019; Citro et al., 2020; Citro et al., 2022) and in Supplementary Table S1. Cells were cultured in Dulbecco’s Modified Eagle’s Medium (DMEM) with stable glutamine, sodium pyruvate (EuroClone), 10% fetal bovine serum (FBS), antibiotics (250 μM/ml penicillin and 25 μM/ml streptomycin), and non-essential amino acids (Lonza), in a humidified incubator with 5% CO_2_ atmosphere at 37°C. Adult human epidermal keratinocytes (HKs) were prepared and maintained as previously described (Mattoscio et al., 2017). Fresh tumors and adjacent normal tissue samples from HNC patients were collected and processed as described in (Citro et al., 2022). Samples were collected from eight HPV-negative and four HPV-positive HNC patients. Six samples were collected from the larynx, three from the oral cavity and the tongue, and three from the oropharynx.

### Immunohistochemistry staining

Tissue samples were formalin-fixed and paraffin-embedded (FFPE) according to established procedures (Crosta et al., 2021). Primary antibodies, anti-UBC9, and anti-E-cadherin were incubated overnight at 4°C. DAKO Envision Kit was utilized, and after the chromogenic visualization step using the 3,3′-diaminobenzidine (DAB) chromogen, slides were counterstained with hematoxylin and coverslipped. Images were generated using a bright field microscope Leica DM6 and semi-quantified by pathologist visual scoring of staining. Lymphocytes staining was used as internal control.

### Cell lysis and western blot

Cells were lysed in a sodium dodecyl sulfate (SDS) lysis buffer: a 1:3 mixture of buffer I [5% SDS, 0.15M Tris-HCl (pH 6.8), 30% glycerol] and buffer II [25 mM Tris-HCl (pH 8.3), 50 mM NaCl, 0.5% NP-40, 0.1% SDS, 1 mM EDTA], containing 0.5 mM N-ethylmaleimide and protease inhibitors. After lysis, an equal amount of protein for each sample was resuspended in denaturing sample loading buffer, separated on SDS-PAGE gel and immunoblotted with the indicated antibodies (Supplementary Table S2). Membranes were then incubated with the appropriate horseradish peroxidase (HRP) secondary antibodies and the signal was acquired with Chemidoc (Bio-Rad). Densitometric analysis of the intensity of the protein bands was preformed using ImageJ software (National Institutes of Health) and standardized to the housekeeper levels.

### RT-qPCR

RNA was extracted from cells with the Quick-RNA MiniPrep kit (Zymo research). cDNA was generated by reverse transcription-PCR with reverse transcriptase (NEB). Relative levels of specific mRNAs were determined with the fast SYBR Green detection chemistry system (NEB) using a QuantStudio 6 Fast Real-Time PCR system (ThermoFisher).

### Immunoprecipitation

HNC cell lines were lysed in Tris-HCl lysis buffer (50 mM Tris-HCl pH 7.6, 150 mM NaCl, 0.8% NP-40, 10% glycerol, 1 mM EDTA, proteases inhibitors, 0.5 mM NEM). Immunoprecipitation was performed using protein extracts incubated with the indicated antibodies o/n at 4°C. Samples were loaded on SDS-PAGE gel and immunoblotted with the indicated antibodies. The total extracts (input) were loaded as control.

### Transduction, transfection, and plasmids

PLXSN HPV16E6/E7 and inducible shUBC9 pTRIPZ lentiviral vector (OpenBiosystem) were previously described (Mattoscio et al., 2017; Citro et al., 2013). HNC cell lines were transfected with Lipofectamine 2000 (Thermo Fisher Scientific), following manufacturers’ instruction with siRNA oligos: siLuciferase (siLUC), siE-cadherin (5′-CAG​ACA​AAG​ACC​AGG​ACU​ATT-3′), or siSUMO1 (Santa Cruz Biotechnology).

### Confocal microscopy

Cells were seeded on coverslips and fixed with 4% PFA. Anti-E-cadherin (rabbit) and anti-β-catenin (mouse) were incubated o/n at 4°C. Goat anti-rabbit Alexa 488 and donkey anti-mouse Alexa 647 were used as secondary antibody and nuclei were stained with DAPI. Coverslips were then mounted and pictures were taken using a Leica SP8 AOBS confocal microscope. ImageJ JACoP plugin was used to measure colocalization.

### Statistical analysis

RSEM normalized expression data were extracted from the TCGA PanCancer Atlas on the cBioPortal (Cerami et al., 2012; Gao et al., 2013). Patient samples were grouped as HPV+ and HPV−. This resulted in 72 HPV+ and 410 HPV−samples with data available for the HNC gene expression analysis. Boxplot comparisons of gene expression and statistical analysis were performed using unpaired *t* test or ANOVA multiple comparison with GraphPad Software (San Diego, California, United States).

## Results

### UBC9 expression increases in HNC samples compared to normal tissues in both nucleus and cytosol

**FIGURE 1:**
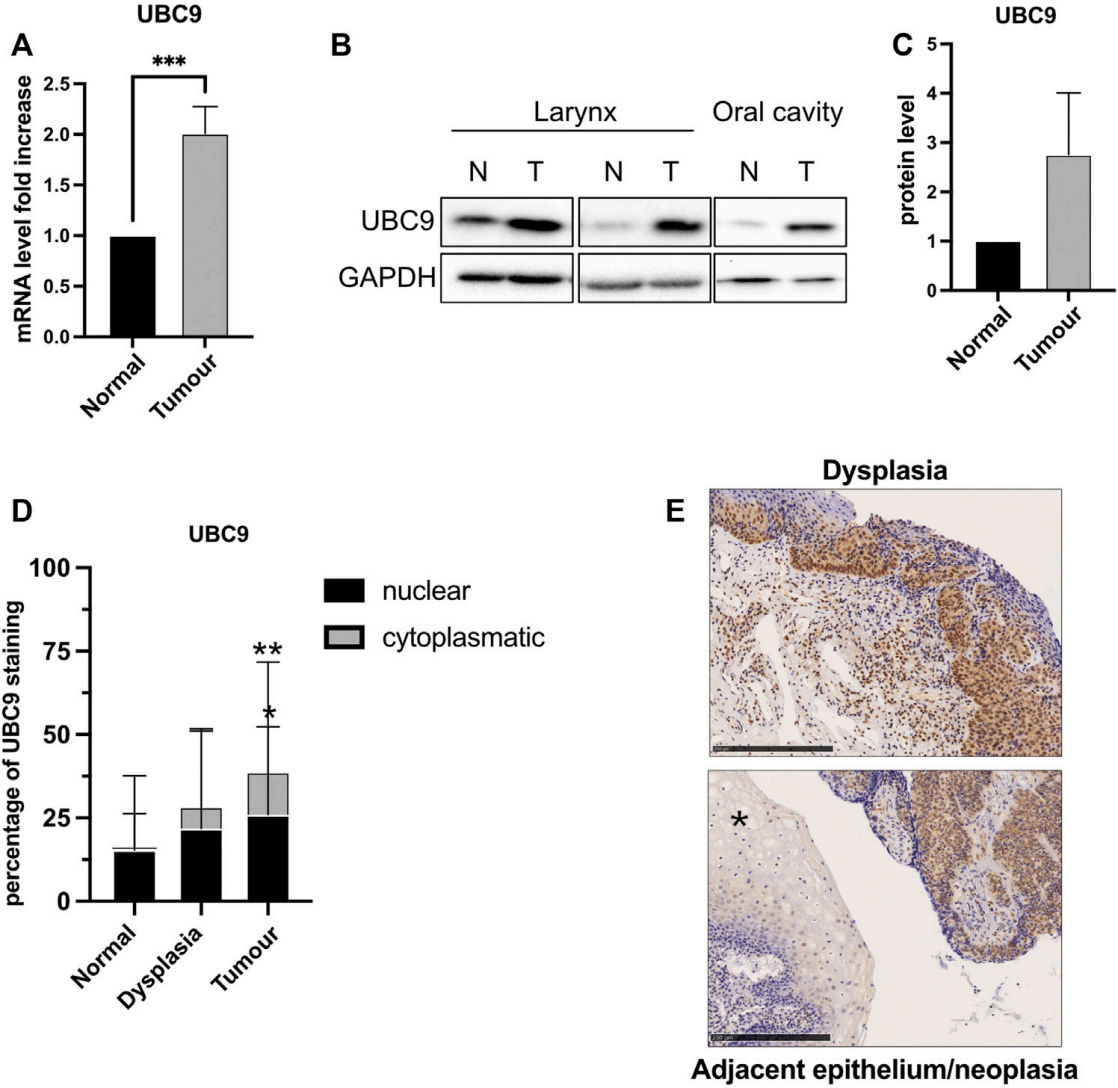
Analysis of UBC9 expression in normal and tumoral HNC tissues. **(A)** Total RNAs from frozen tissue samples of HNC patients (eight HPV-negative and four HPV-positive) were isolated for RT-qPCR and UBC9 expression which were reported as means ± SEM of fold changes. ***, *p* < 0.001 (unpaired *t* test). **(B)** Protein fraction was extracted from frozen tissue samples of HPV-negative HNC patients and analyzed by immunoblotting with the indicated antibodies. **(C)** Graph showing means ± SEM of optical density analysis from immunoblotting of UBC9 expression from eight frozen HNC tissue samples. **(D)** Percentage of UBC9 positivity in nucleus and cytoplasm in dysplastic, tumoral, and matched adjacent mucosa OPC specimens; *, *p* < 0.05, **, *p* < 0.01 (two-way ANOVA multiple comparison test). *n* = 105 (tumor, 56 HPV-negative and 49 HPV-positive), *n* = 36 (dysplasia, 21 HPV-negative, and 15 HPV-positive), *n* = 124 (adjacent mucosa, 84 HPV-negative, and 40 HPV-positive). **(E)** Representative IHC images of FFPE stained with anti-UBC9 antibody from normal (*) and neoplastic OPC tissues (right side of the image) and from high-grade dysplasia, as indicated. Scale bars = 250 μm.

The expression of UBC9 is linked to tumorigenesis and increases in different tumors (Lee et al., 2017; Mattoscio, 2015; Mattoscio et al., 2017). We also observed an increased expression of both mRNA (Figure 1A) and protein (Figures 1B,C) UBC9 levels in fresh HNC tissue samples, compared to normal adjacent tissues. This was confirmed by IHC in HNC tissue samples. As Figure 1D shows, UBC9 levels increased from normal tissue to dysplasia, and even more in neoplastic OPC tissue samples. Interestingly, as shown in Figure 1E, high-grade dysplasia and neoplastic tissues presented an intense and diffuse UBC9 staining in both nucleus and cytoplasm, whereas normal adjacent epithelium showed a really faint UBC9 staining.

### UBC9 binds to E-cadherin preferentially in HPV-positive HNC cell lines and loss of SUMO1 affects cell migration only in HPV-negative HNC cell lines

**FIGURE 2:**
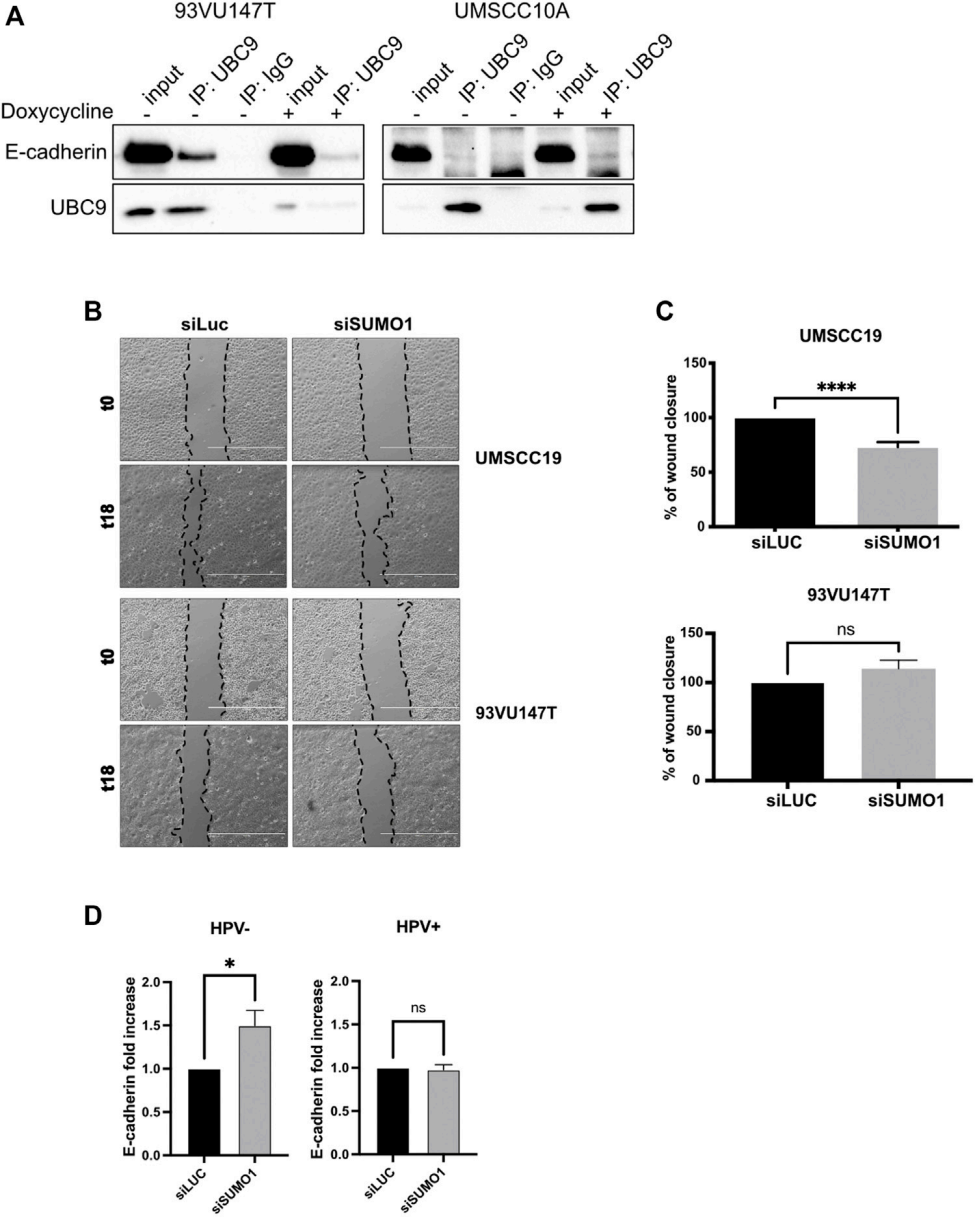
UBC9 interaction with E-cadherin and its transcriptional regulation. **(A)** HPV-positive 93VU147T and HPV-negative UMSCC10A cell lines were transduced with an inducible shUBC9 lentiviral vector, and treated or not with doxycycline for 72 h. Cells were then harvested and whole cell extracts were subjected to anti-UBC9 immunoprecipitation (IP). Immunoblotting with the indicated antibodies was then performed and whole cell extracts were used as input controls. **(B)** Phase-contrast pictures of scratch wound healing assay performed on HPV-positive 93VU147T and HPV-negative UMSCC19, transfected for 72 h with SUMO1 or LUC siRNA at time 0 and 18 h after the scratch. **(C)** Graph represented the percentage of wound closure (means ± SEM) of SUMO1 siRNA cells as compared with LUC siRNA transfected; ****, *p* < 0.0001, ns, not significant (unpaired *t* test). **(D)** HPV-positive HNC cell lines (UMSCC47, UPCISCC154, 93VU147T) and HPV-negative (UMSCC10A, UMSCC19, UMSCC23) cell lines were transfected for 72 h with specific siRNA against SUMO1 or Luciferase as control. After transfection, total RNAs were isolated for RT-qPCR and reported as means ± SEM of fold changes of at least three independent experiments; *, *p* < 0.05, ns, not significant (unpaired *t* test).

Binding to UBC9 is required for the correct localization at the plasma membrane of different proteins (Collec et al., 2007; Xiong et al., 2017). As shown in Figure 2A and Supplementary Figure S1A, UBC9 was able to specifically interact with E-cadherin preferentially in HPV-positive HNC cell lines, and only in a small portion in HPV-negative cell lines, in accordance with our previous findings showing that HPV upregulates UBC9 expression (Mattoscio et al., 2017). E-cadherin has been previously found to be SUMOylated preferentially by SUMO1 (Ha et al., 2016) and we also confirmed this by co-immunoprecipitation (Supplementary Figure S1B). Thus, we evaluated the effect of SUMO1 knock-down (Supplementary Figure S1C) on migration in both HPV-positive and HPV-negative HNC cell lines. As Figures 2B,C show, we noticed that SUMO1 silencing was decreasing cell motility only in HPV-negative HNC cell lines, probably through an indirect regulation of E-cadherin transcription (Figure 2D). Interestingly, SUMO1 knock-down did not affect E-cadherin transcription in HPV-positive HNC cell lines (Figure 2D), with no significant change in cell migration rate (Figures 2B,C). All these data suggest that E-cadherin is differentially regulated by SUMO in HPV-positive compared to HPV-negative cell lines.

### SUMOylation protects E-cadherin from cleavage in HPV-positive HNC cell lines

**FIGURE 3:**
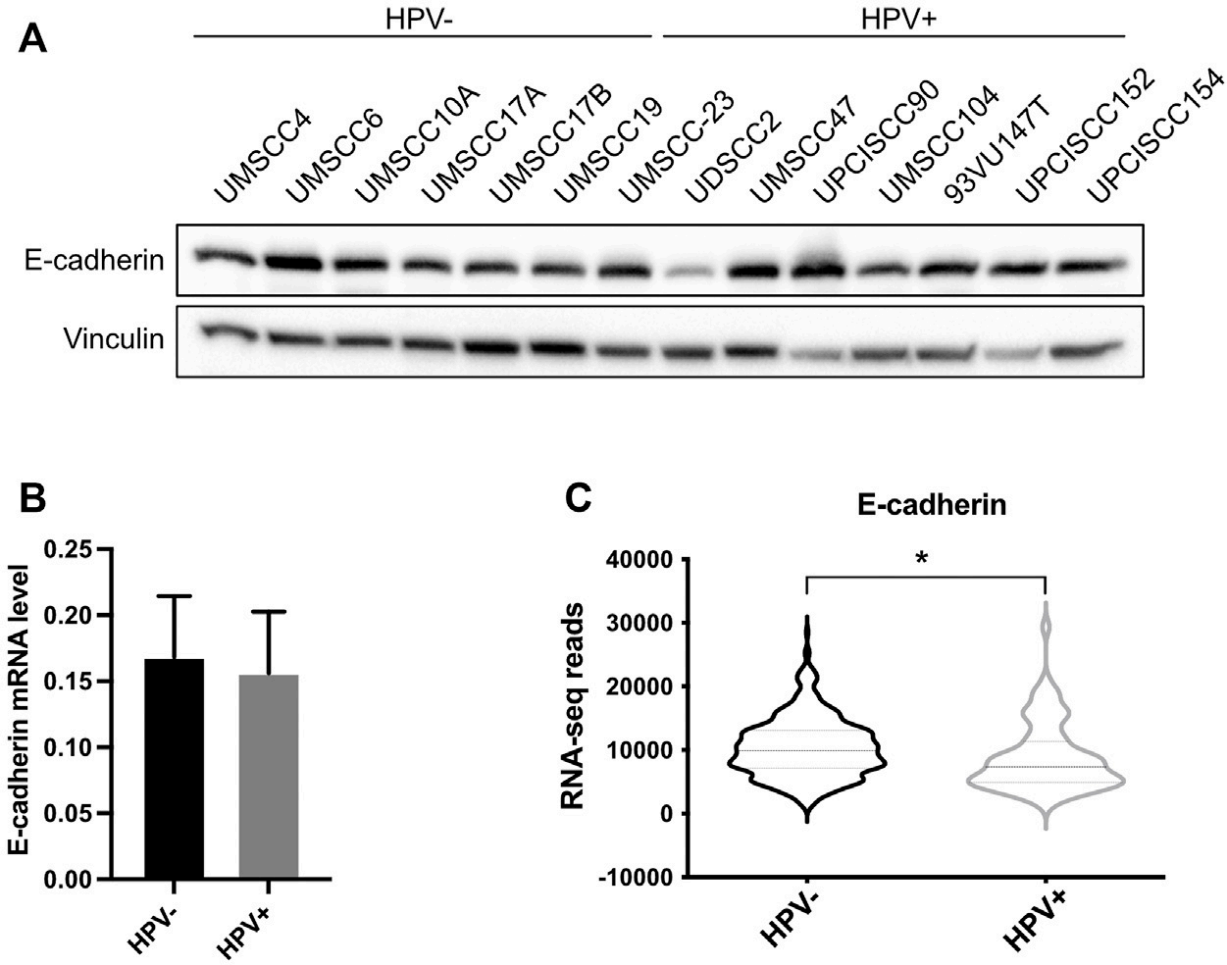
Analysis of E-cadherin expression in HPV-positive and HPV-negative HNC. **(A)** HNC cell lines were lysed and analyzed by immunoblotting with the indicated antibodies (FL, full-length). **(B)** Total RNAs from HNC cell lines were isolated for RT-qPCR. E-cadherin expression was normalized to RpP0 and shown as means ± SEM of at least three independent experiments. **(C)** Normalized RNA-seq data were extracted from TCGA database and divided in HPV-positive and HPV-negative HNC cohorts. *, *p* < 0.05 (unpaired *t* test).

**FIGURE 4:**
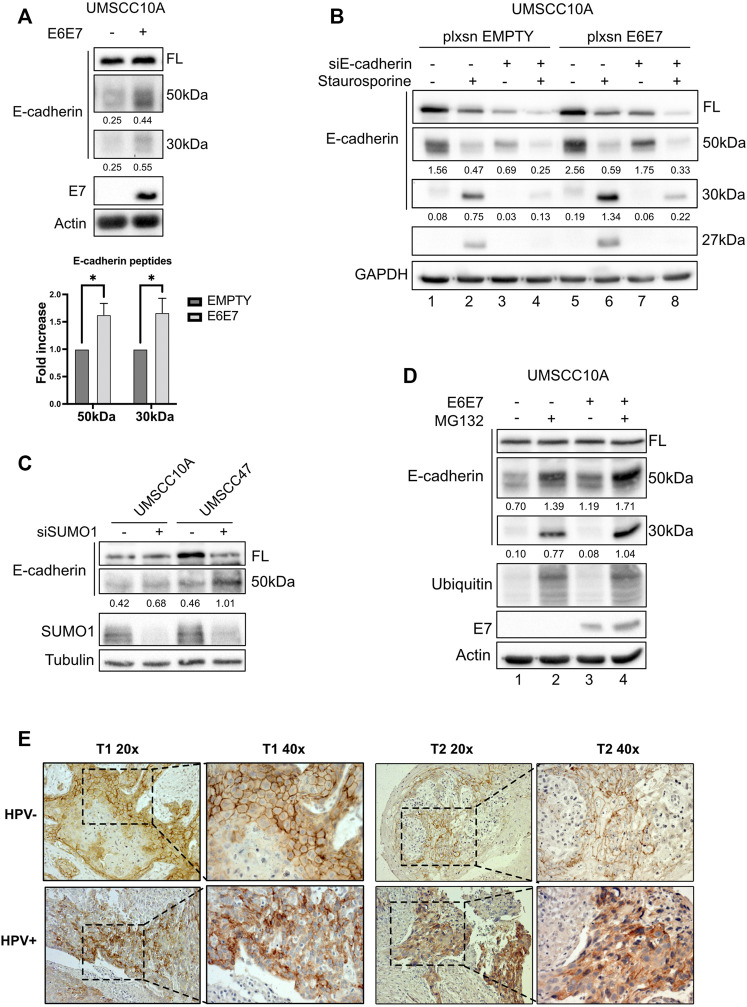
HPV16 and SUMO1 regulation of E-cadherin cleavage and localization. **(A)** UMSCC10A cells were transduced with empty or HPV16E6/E7 recombinant retroviral vectors. Lysates were analyzed by immunoblotting with the indicated antibodies. Graph represented densitometric analysis and reported as means ± SEM of fold changes, *, *p* < 0.05 (two-way ANOVA multiple comparison test). *n* = 7.**(B)** UMSCC10A cells transduced with empty or HPV16E6/E7 recombinant retroviral vectors, were transfected with specific siRNA against E-cadherin or Luciferase as control. 72 h after transfection, cells were treated or not for 6 h with 1 μM staurosporine, lysed and analyzed by immunoblotting with the indicated antibodies (FL, full-length). Densitometric analysis was performed and optical density values normalized to loading control were reported below each band. **(C)** HPV-positive HNC cell lines (UMSCC47) and HPV-negative (UMSCC10A) cell lines were transfected for 72 h with specific siRNA against SUMO1 or Luciferase as control. Cells were then lysed and analyzed by immunoblotting with the indicated antibodies. **(D)** UMSCC10A cells were transduced with empty or HPV16E6/E7 recombinant retroviral vectors. Cells were then seeded and treated with 10 μM MG132 for 16 h, lysed and analyzed by immunoblotting with the indicated antibodies. **(E)** Representative IHC images of FFPE from tumoral HN oropharyngeal HPV-positive or negative tissues, as indicated, stained with anti-E-cadherin antibody, presented at two different magnifications (40x and 20x).

Since UBC9 shows more affinity in binding E-cadherin in HPV-positive cell lines and since lack of SUMOylation did not affect E-cadherin transcription, we asked whether SUMO was influencing the expression of E-cadherin at post-transcriptional level in HPV-positive cell lines. We first assessed the expression of E-cadherin in our panel of cell lines. As shown in Figures 3A,B, we could not detect any significant difference in the expression of E-cadherin at both mRNA and protein level between HPV-positive and negative HNC cell lines, and only a slight decrease in HPV-positive compared to HPV-negative HNC samples from TCGA dataset (Figure 3C). E-cadherin is known to undergo protein cleavage at the intracellular domain upon the activation of different proteolytic events (Steinhusen et al., 2001; Yoo et al., 2012). We transduced E6/E7 in the HPV-negative cell line UMSCC10A (Supplementary Figure S1D) and performed Western blot analysis using an antibody that recognizes the cytoplasmic domain of E-cadherin. As Figure 4A shows, HPV oncoproteins induced the formation of two different E-cadherin peptides at 50 and 30 kDa. We confirmed by E-cadherin silencing (Supplementary Figure S1E) that the 50 and 30 kDa products were specific products of E-cadherin cleavage (Figure 4B compared lane 1 to lane 3 and lane 5 to lane 7). We then treated UMSCC10A cells with staurosporine, which is known to induce caspase-dependent E-cadherin cleavage (Yoo et al., 2012). As shown in Figure 4B, staurosporine was able to cleave E-cadherin into two main products at 30 and 27 kDa, reducing both full length and the 50 kDa bands of E-cadherin (Figure 4B compared lane 1 to lane 2 and lane 5 to lane 6), suggesting that the 27 kDa product derived from the subsequent cleavage of the 50 kDa peptide. Interestingly, SUMO1 knock-down increased E-cadherin cleavage preferentially in HPV-positive cell lines (Figure 4C), hinting that in HPV-positive cell lines UBC9 and SUMO1 are needed to maintain E-cadherin localization at the plasma membrane.

### Cleaved E-cadherin undergoes protein degradation

It has been previously shown that E-cadherin products derived from cleavage are degraded by an intracellular proteolytic pathway (Ito et al., 1999). We then treated UMSCC10A cells, transduced or not with E6/E7 oncoproteins, with the proteasome inhibitor MG132. As shown in Figure 4D, MG132 increased the 50 kDa and the 30 kDa products in UMSCC10A (compare lane 1 to lane 2) and even more in cells transduced with E6/E7 (compare lane 3 to 4), without changing the full-length form, confirming that E-cadherin cleaved products undergo protein degradation.

### E-cadherin localization at the plasma membrane is disrupted in HPV-positive HNC tumors

We finally investigated whether the increased E-cadherin proteolysis detected in HPV-positive compared to HPV-negative HNC cell lines, was detectable also in tumor samples. We thus screened for IHC different HNC specimens derived from HPV-positive and HPV-negative HNC and, as shown in Figure 4E, we found that in HPV-negative HNC, E-cadherin was preferentially present at the cell surface; HPV-positive HNC showed an intracellular staining of E-cadherin. Moreover, as shown in Supplementary Figure S1F, we confirmed that also in human keratinocytes transduced with HPV16 E6/E7, the presence of the two oncoproteins decreased the integrity of adherent junctions, leading to the dissociation of E-cadherin from β-catenin, which is a consequence of E-cadherin cleavage (Ito et al., 1999). These data show that cell surface E-cadherin is reduced in HPV-positive HNC, and this can be explained by its increased proteolysis.

## Discussion

Confirming our previous data (Mattoscio et al., 2017), here we show that in HNC, UBC9 expression increases with the progression of the tumor. Interestingly, we also noticed that together with the increase of nuclear UBC9 staining, in dysplastic and even more in tumoral tissue, UBC9 appeared in the cytoplasm. Previous data are consistent with these results, as UBC9 protein has been found in the cytosol, nucleus, membranes, and endoplasmic reticulum in a variety of cell types (Gupta et al., 2014), and UBC9 binding has been shown to be crucial for the correct localization at the plasma membrane of different proteins (Collec et al., 2007; Xiong et al., 2017). It has been previously shown that, both UBC9 and SUMO1, promote cell migration, increasing invasion and metastasis in different tumors (Castillo-Lluva et al., 2010; Zhu et al., 2010; LI et al., 2013). Interestingly, here we show that loss of SUMO1 confers a decreased mobility only in HPV-negative HNC cell lines with change in E-cadherin transcription, consistent with the role of the SUMO pathway in affecting E-cadherin transcription through secondary effects (Bogachek et al., 2015). The loss of intercellular adhesion mediated by E-cadherin is a critical change that occurs during the evolution of cancer into invasive disease. HPV16 E6/E7 oncoproteins have been shown to negatively regulate E-cadherin by different mechanisms (Laurson et al., 2010; Caberg et al., 2008). Lower E-cadherin expression has been associated with a higher risk of distant metastasis (Lefevre et al., 2017), but different studies could not find any association between E-cadherin expression and HPV status in OPC (Lefevre et al., 2017; Mohamed et al., 2020). We also could not detect any differences in the expression of full-length E-cadherin between HPV-positive and HPV-negative HNC cell lines and only a small difference in the mRNA expression of E-cadherin between HPV-positive and HPV-negative HNC tissue samples. Interestingly, HNC cell lines transduced with HPV16 E6/E7 showed an increased expression of cleaved E-cadherin peptides, which are targets for proteolytic degradation, in accordance with the evidences showing that HPV increases the activity and the expression of both caspases and metalloproteinases (Herbster et al., 2018; Manzo-Merino et al., 2014). On the other side, HPV is known to increase UBC9 expression (Mattoscio et al., 2017) and this seems to promote the selective binding of E-cadherin to UBC9 in HPV-positive HNC cell lines and protects E-cadherin from its proteolysis. All these data show a different behavior of the SUMO/UBC9 pathway between HPV-positive and negative HNC cell lines. In HPV-negative cells, lack of the SUMO/UBC9 pathway decreases cell motility, as seen in other cancers, and this seems in part due to an increased transcription of E-cadherin. On the contrary, in HPV-positive HNC cell lines, UBC9 is able to interact with E-cadherin and this interaction somehow protects E-cadherin from its HPV-mediated proteolysis. Many evidences indicate that peptides derived from the proteolysis of E-cadherin might promote tumor growth, survival, and motility. In this study, we found a role for the SUMO/UBC9 pathway in interfering with HPV-mediated E-cadherin cleavage, which might transform this tumor suppressor into an oncogenic factor. Moreover, we revealed for the first time, in accordance with previous findings (Hubert et al., 2005; Rodríguez-Sastre et al., 2005) showing an increased cytoplasmatic E-cadherin staining in the progression of cervical cancer, that in HPV-positive HNC specimens E-cadherin staining is prevalently cytoplasmatic compared to HPV-negative HNC. These data uncover an important molecular feature of HPV-positive HNC that might improve the diagnosis and therapy of these tumors.

## Data Availability

The original contributions presented in the study are included in the article/Supplementary Material, further inquiries can be directed to the corresponding authors.
