# Supplementary material for: The UBC9/SUMO pathway affects E-cadherin cleavage in HPV-positive head and neck cancer
Source: Front Mol Biosci. 2022 Aug 12;9:940449. doi: 10.3389/fmolb.2022.940449 (PMC9411811; doi:10.3389/fmolb.2022.940449)
Supplement: Supplementary file 1 [file DataSheet1.pdf]

Supplementary Material

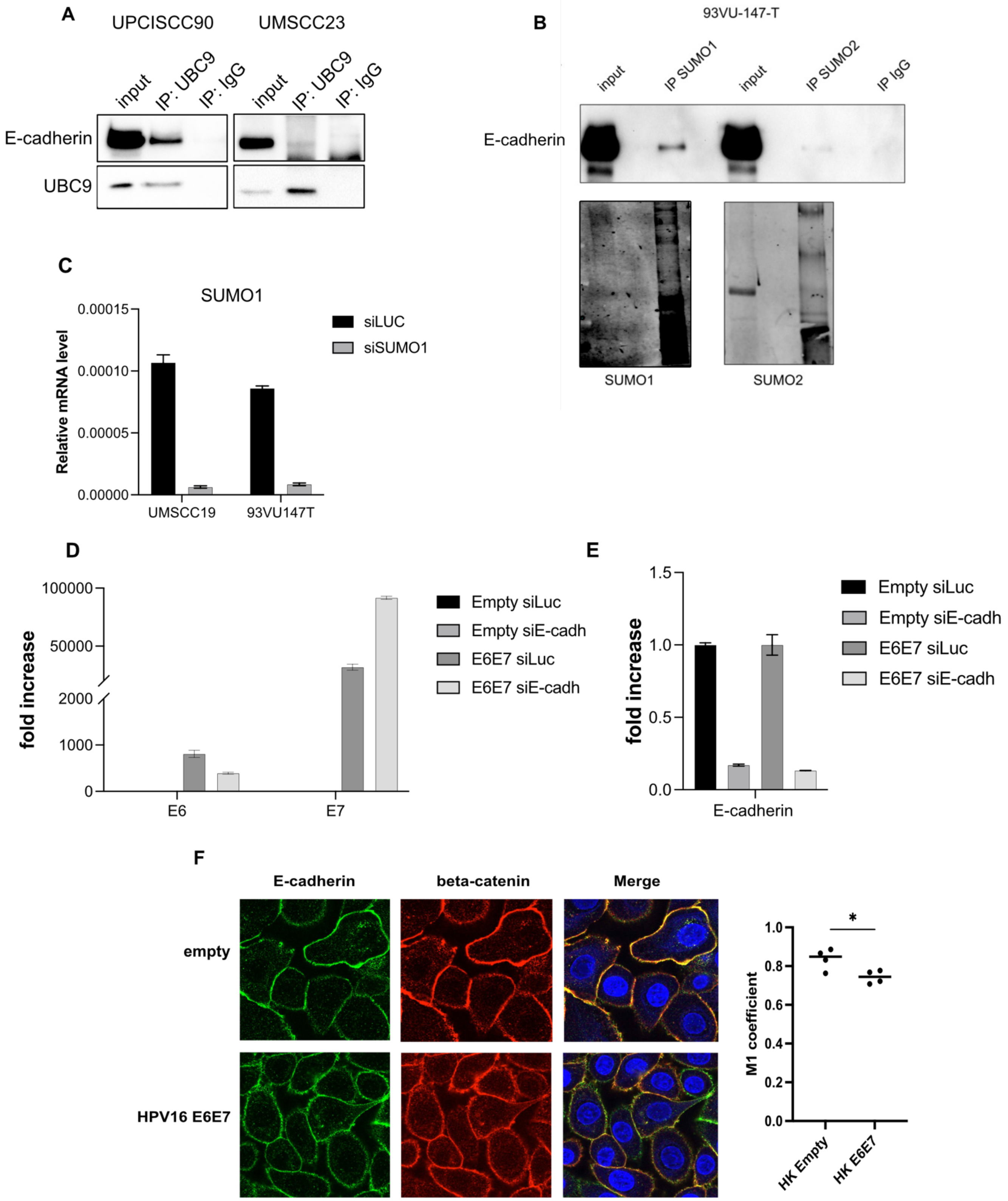

Supplementary Figure. (A) HPV-positive UPCISCC90 and HPV-negative UMSCC23 cell lines were harvested and whole cells extracts were subjected to anti-UBC9 immunoprecipitation (IP). Immunoblotting with the indicated antibodies was then performed and whole cell extracts were used as input controls. (B) HPV-positive 93VU147T cell line was harvested and whole cells extracts were subjected to anti-SUMO1 or SUMO2 immunoprecipitation (IP). Immunoblotting with the indicated antibodies was then performed and whole cell extracts were used as input controls. (C) HPV-positive 93VU147T and HPV-negative UMSCC19 were transfected for 72h with specific siRNA against SUMO1 or Luciferase as control. After transfection total RNAs were isolated for RT-qPCR and SUMO1 expression was normalized to RpP0 and shown as means ± SD. (D, E) UMSCC10A cell line was transduced with empty or HPV16E6/E7 recombinant retroviral vectors. After selection with G418 cells were transfected with specific siRNA against E-cadherin or Luciferase as control. 72h after transfection total RNAs were isolated for RT-qPCR and reported as means ±SD of fold changes. (F) Human Keratinocytes (HK) were transduced with empty or HPV16E6/E7 recombinant retroviral vectors. After selection with G418 cells were seeded on coverslips and after 24h fixed and stained with the indicated antibodies for Immunofluorescence. Coverslips were mounted and visualized using a Leica SP8 AOBS confocal microscopy. E-cadherin (green) and β-catenin (red) colocalization was evaluated in 4 different images and measured by Mander's coefficient (M1) as shown in the graph. *, $P < 0.05$ (unpaired t test).

CELL LINE	HPV STATUS	SUBSITE	SEX	ORIGIN
UMSCC4	Negative	Oropharynx	Female	(Brenner *et al.*, 2010) (Zhao *et al.*, 2010)
UMSCC6	Negative	Oropharynx	Male	(Brenner *et al.*, 2010) (Zhao *et al.*, 2010)
UMSCC10A	Negative	Larynx	Male	(Brenner *et al.*, 2010) (Zhao *et al.*, 2010)
UMSCC17A	Negative	Larynx	Female	(Carey *et al.*, 1989)
UMSCC17B	Negative	Larynx	Female	(Carey *et al.*, 1989)
UMSCC19	Negative	Oropharynx	Male	(Brenner *et al.*, 2010), (Zhao *et al.*, 2010)
UMSCC23	Negative	Larynx	Female	(Brenner *et al.*, 2010)
UDSCC2	Positive	Hypopharynx	Male	(Balló *et al.,* 1999)
UMSCC47	Positive	Oropharynx	Male	(Brenner *et al.*, 2010), (Zhao *et al.*, 2010)
UPCISCC90	Positive	Oropharynx	Male	(Ragin, Reshmi and Gollin, 2004)
UMSCC104	Positive	Oral cavity	Male	(Tang *et al.,* 2011)
UPCISCC152	Positive	Hypopharynx	Male	(Kalu *et al.*, 2017), (White *et al.*, 2007)
UPCISCC154	Positive	Oral cavity	Male	(White *et al.*, 2007)
93VU147T	Positive	Oral cavity	Male	(Steenbergen *et al.*, 1995)

Supplementary Table1. List of used HNC cell lines.

antibody	Company	Clone
anti-UBC9	Novus Biologicals	Catalog n: NBP1-86887
anti-E-cadherin	Cell Signaling Technology	Catalog n: 3195
anti-GAPDH	Abcam	6C5
anti-SUMO1	SIGMA	Catalog n: S8070
anti-vinculin	SIGMA	hVIN1
anti-tubulin	SIGMA	DM 1A
anti-actin	Novus Biologicals	AC-15
anti-E7(HPV16)	Santa Cruz Biotechnology	NM2
anti-ubiquitin	Santa Cruz Biotechnology	P4D1
anti-p53	Santa Cruz Biotechnology	DO-1
anti-β-catenin	BD Bioscences	14/Beta-Catenin

Supplementary Table2. List of used antibodies.

References

Balló, P Koldovsky, T Hoffmann, V Balz, B Hildebrandt, C D Gerharz, H.B. (1999) 'Establishment and characterization of four cell lines derived from human head and neck squamous cell carcinomas for an autologous tumor-fibroblast in vitro model', *Anticancer Research*, 19(5B), pp. 3827–36.

Brenner, J.C. *et al.* (2010) 'Genotyping of 73 UM-SCC head and neck squamous cell carcinoma cell lines', *Head and Neck*, 32(4), pp. 417–426. doi:10.1002/hed.21198.

Carey, T.E. *et al.* (1989) ‘Characterization of Human Laryngeal Primary and Metastatic Squamous Cell Carcinoma Cell Lines UM-SCC-17A and UM-SCC-17B’, *Cancer Research*, 49(21), pp. 6098–6107.
Kalu, N.N. *et al.* (2017) ‘Genomic characterization of human papillomavirus-positive and -negative human squamous cell cancer cell lines’, *Oncotarget*, 8(49), pp. 86369–86383. doi:10.18632/oncotarget.21174.
Ragin, C.C.R., Reshmi, S.C. and Gollin, S.M. (2004) ‘Mapping and analysis of HPV16 integration sites in a head and neck cancer cell line’, *International Journal of Cancer*, 110(5), pp. 701–709. doi:10.1002/ijc.20193.
Steenbergen, R.D.M. *et al.* (1995) ‘Integrated Human Papillomavirus Type 16 and Loss of Heterozygosity at 11q22 and 18q21 in an Oral Carcinoma and Its Derivative Cell Line’, *Cancer Research*, 55(22), pp. 5465–5471.
Tang, Samantha J. Hauff, John H. Owen, Martin P. Graham, Michael J. Czerwinski, J.J.P. and Heather Walline, Silvana Papagerakis, Jay Stoerker, Jonathan B. McHugh, Douglas B. Chepeha, Carol R. Bradford, Thomas E. Carey, E.P. (2011) ‘UM-SCC-104: A New human papillomavirus-16–positive cancer stem cell–containing head and neck squamous cell carcinoma cell line’, *Wiley Online Library* [Preprint]. doi:10.1002/hed.21962.
White, J.S. *et al.* (2007) ‘The influence of clinical and demographic risk factors on the establishment of head and neck squamous cell carcinoma cell lines’, *Oral Oncology*, 43(7), pp. 701–712. doi:10.1016/j.oraloncology.2006.09.001.
Zhao, M. *et al.* (2010) ‘Assembly And Initial Characterization Of A Panel Of 85 Genomically Validated Cell Lines From Diverse Head And Neck Tumor Sites’, 17(23), pp. 7248–7264. doi:10.1158/1078-0432.CCR-11-0690.Assembly.
